# Hospital visit pattern and its effect on reperfusion time and clinical outcomes in ST-segment elevation acute myocardial infarction

**DOI:** 10.1186/cc13380

**Published:** 2014-03-17

**Authors:** J Park, Y Kim, D Shin, W Kim, S Lee, J Son

**Affiliations:** 1 Yeungnam University Hospital, Daegu, South Korea

## Introduction

The reperfusion time is critical in ST-segment elevation myocardial infarction (STEMI), and it makes a difference to clinical outcomes. This study was designed to investigate the hospital visit pattern and its effect on reperfusion time and clinical outcomes of STEMI patients.

## Methods

A total of 199 STEMI patients were registered in this study from three university hospitals in Kyungsang-do area, Korea, and were divided into two groups; group 1 (*n *= 69) who directly visited the hospitals capable of percutaneous coronary intervention (PCI), and group 2 (*n *= 130) who first visited local hospitals and then transferred to PCI-capable hospitals. We analyzed the estimated distance and time to the hospitals using a driving navigation system, elapsed time from chest pain to primary PCI hospitals, chest pain to reperfusion time, and in-hospital outcomes.

## Results

There was no difference in first medical contact time between groups 1 and 2. But the time from chest pain to PCI hospital was shorter in group 1 (206.2 ± 268.5 vs. 370.3 ± 415.2 minutes, *P *= 0.001). Sixty patients in group 1 and 108 patients in group 2 underwent reperfusion therapy (*P *= 0.473). The chest pain to reperfusion time was shorter in group 1 (294.6 ± 255 vs. 397.2 ± 341.9 minutes, *P *= 0.045). The difference in estimated time by navigator and actual hospital visit time was also shorter in group 1 (194.2 ± 269.9 vs. 321.6 ± 411.0 minutes, *P *= 0.009). In-hospital mortality was higher in group 2 (0 vs. 4.6%, *P *= 0.094).

## Conclusion

A primary visit to a local hospital was associated with longer reperfusion time and was associated with higher mortality. Therefore, the reperfusion time could be reduced by patient education and management of the community healthcare system.

